# Chicken CRTAM Binds Nectin-Like 2 Ligand and Is Upregulated on CD8^+^ αβ and γδ T Lymphocytes with Different Kinetics

**DOI:** 10.1371/journal.pone.0081942

**Published:** 2013-12-10

**Authors:** Maria Zechmann, Sven Reese, Thomas W. Göbel

**Affiliations:** 1 Institute for Animal Physiology, Department of Veterinary Sciences, University of Munich, Munich, Germany; 2 Institute for Anatomy, Histology and Embryology, Department of Veterinary Sciences, University of Munich, Munich, Germany; University of Nebraska Medical center, United States of America

## Abstract

During a search for immunomodulatory receptors in the chicken genome, we identified a previously cloned chicken sequence as CRTAM homologue by its overall identity and several conserved sequence features. For further characterization, we generated a CRTAM specific mab. No staining was detectable in freshly isolated cell preparations from thymus, bursa, caecal tonsils, spleen, blood and intestine. Activation of splenocytes with recombinant IL-2 increased rapid CRTAM expression within a 2 h period on about 30% of the cells. These CRTAM^+^ cells were identified as CD8^+^ γδ T lymphocytes. In contrast, CRTAM expression could not be stimulated on PBL with IL-2, even within a 48 h stimulation period. As a second means of activation, T cell receptor (TCR) crosslinking using an anti-αβ-TCR induced CRTAM on both PBL and splenocytes. While CRTAM expression was again rapidly upregulated on splenocytes within 2 h, it took 48 h to reach maximum levels of CRTAM expression in PBL. Strikingly, albeit the stimulation of splenocytes was performed with anti-αβ-TCR, CRTAM expression after 2 h was mainly restricted to CD8^+^ γδ T lymphocytes, however, the longer anti-TCR stimulation of peripheral blood lymphocytes (PBL) resulted in CRTAM expression on αβ T lymphocytes. In order to characterize the potential ligand we cloned and expressed chicken Necl-2, a member of the nectin and nectin-like family which is highly homologous to its mammalian counterpart. Three independent assays including a reporter assay, staining with a CRTAM-Ig fusion protein and a cell conjugate assay confirmed the interaction of CRTAM with Necl-2 which could also be blocked by a soluble CRTAM-Ig fusion protein or a CRTAM specific mab. These results suggest that chicken CRTAM represents an early activation antigen on CD8^+^ T cells which binds to Necl-2 and is upregulated with distinct kinetics on αβ versus γδ T lymphocytes.

## Introduction

CD8^+^ T cells and natural killer (NK) cells display a number of receptors that are important in the recognition of target cells. One group of receptors expressed by both cell types belongs to the immunoglobulin superfamily (IgSF) and includes CD226 (DNAM-1), TIGIT (WUCAM), CD96, and CRTAM (CD355) [1], [2]. These receptors bind to members of the nectin and nectin-like family, which are IgSF members, too and mediate Ca^2+^ independent homophilic and heterophilic adhesion. They are also important for adhesion between neighboring epithelial cells.

CRTAM was initially identified in a study devised to characterize upregulated genes in NK-T cells and received its name as “class I-restricted T cell-associated molecule” to denote its restricted expression pattern on CD8^+^ T cells. It has been further characterized as an important activation marker for both CD8^+^ T cells and NK-T cells [3]. In man, CRTAM expression is observed on activated CD8^+^ T cells, as well as on natural killer (NK) cells and a small subset of CD4^+^ T cells [3], [4]. As a type I transmembrane protein CRTAM is composed of an amino-terminal Ig-like domain of the variable type (V-type), and a membrane proximal Ig-like domain of the constant type 1 (C1-type). Its intracytoplasmic domain contains a carboxyl–terminal class I PSD-95/Disc-large/ZO-1 (PDZ)-binding motif, capable of interacting with PDZ domain.

The CRTAM gene is located on human chromosome 11 (11q24.1), whereas the mouse counterpart is located on chromosome 9 (9 21.79 cM), both consisting of 10 exons. Several groups have identified Necl-2 (CADM1, IGSF4, SYNCAM, TSLC1, synCAM1) as a ligand of both murine and human CRTAM [4]–[7]. Engagement of CRTAM promotes cytotoxic effects of NK cells towards Necl-2 expressing tumor cells. Coligation of TCR and CRTAM on CD8 positive T cells causes interferon-γ (IFN-γ) secretion in vitro [4].

Recently, a study highlighted the role of CRTAM expression on Vγ9Vδ2 T cells after TCR triggering. Although this T cell subpopulation exerted neither cytotoxicity nor cytokine secretion, an interaction of CRTAM on these cells with Necl-2 bearing tumor cells induced CRTAM down regulation and cell death of the Vγ9Vδ2 T cells, thus revealing the CRTAM involvement in the survival of activated Vγ9Vδ2 T cells [8].

Aside from the presence on cells of the immune system and its immunological functions, CRTAM is also involved in cell-cell adhesion due to its expression in epithelial cells [7] and has a role in the transcytosis of neural stem cells across brain vascular endothelial cells [9].

In 2000, a novel chicken receptor was characterized as member of the IgSF [10]. Since mRNA could only be detected in mitogen-treated thymic and splenic cells as well as in embryonic cells at developmental time points known for T cell clonal expansion it was designated as cTADS (chicken thymic activation and developmental sequence).

The characterization of IgSF families in the chicken with immunomodulatory capacity has been instrumental to gain novel insights into the phylogeny, tissue distribution and function of various IgSF immune receptors [11]–[13]. Therefore, during an attempt to find additional families, sequence analyses revealed that cTADS is the chicken CRTAM homologue. We now demonstrate using a novel CRTAM specific mab that avian CRTAM expression is restricted to activated CD8^+^ T cells. Although absent from resting cells, stimulation with IL-2 or anti-αVβ1-TCR leads to a rapid CRTAM upregulation on CD8^+^ γδ T cells and at later time points on αβ T cells. Furthermore, we were able to characterize Necl-2 as a ligand for chicken CRTAM. These results point to an important function of CRTAM especially on γδ T cells, a close interaction of αβ and γδ T cells during activation and they finally provide evidence for the high phylogenetic conservation of the adhesion receptor family on lymphocytes that binds to nectins.

## Materials and Methods

### Ethics Statement

All of the experimental procedures were in accordance with institutional, state and federal guidelines on animal welfare. The animal experiments were approved by the committee for the Care and Use of Laboratory animals of the Government of Upper Bavaria, Germany (permit number: 55.2-1-54-2531.6-12.09) and all efforts were made to minimize animal suffering during work.

### Animals

Fertilized eggs of chicken line M11, provided by S. Weigend, Federal Agricultural Research Center, Mariensee, Germany, were hatched at the Institute for Animal Physiology, University of Munich. The birds were raised under conventional conditions and used for experiments at the age of 8–16 weeks. Balb/c mice were purchased from Charles River Wiga GmbH (Sulzfeld, Germany).

### Cloning procedures and sequence analysis

The chicken thymic activation and developmental sequence (cTADS) was originally described in a previous study [10] and will be named CRTAM throughout the paper. For the generation of the constructs, PCR primers for amplification were designed based on the CRTAM and Necl-2 sequence (CRTAM: accession no. NM_001030347.1, Necl-2: accession number XM_417901.3; Table 1). The primers were flanked by sequences recognizing residues of the respective cloning vectors to allow cloning with overlapping ends using the Gibson Assembly™ Master Mix. (New England Biolabs, Ipswich, USA). As templates for CRTAM and Necl-2 amplification, cDNA derived from a chicken thymic cell line (T1-6G5) and chicken brain was used, respectively.

**Table 1:** Oligonucleotides used for cloning.

Number	Sequence	O	Specificity
1718	GGACGATGACGATAAGGAAttcgaaaccataactgta	S	CRTAM
1719	CTGTGCTGGATATCTGCAatccttctccttcttac	AS	CRTAM
1740	GGACGATGACGATAAGggccagaacttgataacag	S	Necl-2
1741	TGGATATCTGCAGAATTcgtccacgctccttattg	AS	Necl-2
1756	AAGCTGGTGCCACGCGaaaccataactgtacagg	S	CRTAM-Ig
1757	GGCATGTGTGAGTTTTGtccttctccttcttactc	AS	CRTAM-Ig

O: orientation indicated as S (sense) and AS (antisense); Capital letters denote regions overlapping with vectors and lower case letters those regions specific for the gene indicated.

After purification with Wizard®SV Gel and PCR Clean-up system (Promega, Mannheim, Germany), the PCR products were linked to a modified pcDNA3.1/V5-His Topo Vector (Invitrogen, Karlsruhe, Germany) containing a N-terminal FLAG epitope tag, the transmembrane domain of chicken CD8α and the cytoplasmic domain of murine CD3ζ [14], [15] using the Gibson Assembly™ Master Mix. In order to generate a soluble CRTAM-Ig fusion protein the primers were generated to amplify the CRTAM extracellular region with overlapping sequences to a vector containing the human Ig signal peptide and the C_H_2, C_H_3 domain of human IgG1 [16] using the Gibson Assembly. All constructs were verified by sequencing (GATC, Konstanz, Germany). The Lasergene software package was used for sequence analyses and Mega5 [17] for the construction of the phylogenetic tree.

### Cell lines, transfection and expression

The chicken CRTAM-FLAG and Necl-2-FLAG constructs were used to establish stable HEK 293 cells and the CRTAM-FLAG was also used to generate a stable BWZ.36 cell line. The human embryonic kidney (HEK) 293 cell line [18] was stably transfected using the Metafectene liposomal transfection reagent according to the manufacturer's protocol (Biontex, Planegg, Germany) for both CRTAM and Necl-2. After 24 h of incubation (37°C, 5% CO_2_) the transfected cells were seeded in a 96-well flat bottom plate and cultured with medium containing 0.8 mg/ml G418 (Biochrom AG, Berlin, Germany) for 2 weeks.

The mouse thymocyte cell line BWZ.36 [19] was stably transfected by electroporation (3×10^6^ cells, 200 V with 950 µF capacitance) with the CRTAM-FLAG expression construct.

Transfected cells were plated in a 96-well flat bottom plate and selected in medium containing 0.8 mg/ml G418 (Biochrom AG, Berlin, Germany) for 10 days at 37°C.

Grown single colonies were analyzed by flow cytometry (FACS CantoII, BD, Heidelberg, Germany) for surface expression of the FLAG epitope.

### Expression of chicken CRTAM-Ig fusion protein and ELISA

The CRTAM-Ig fusion construct was stably transfected into HEK 293 cells using the Metafectene liposomal transfection reagent according to the manufacturer's protocol (Biontex, Planegg, Germany). After 24 h of incubation (37°C, 5% CO_2_) the transfected cells were seeded in a 96-well flat bottom plate and cultured with medium containing 0.8 mg/ml G418 (Biochrom AG, Berlin, Germany) for 2 weeks. The culture supernatants were analyzed for CRTAM-huIg secretion by sandwich ELISA as described [20]. Soluble CRTAM was then affinity-purified on protein G-coupled Sepharose using standard procedures.

### Generation of a monoclonal antibody

For mab generation, Balb/c mice were immunized three times in 3 weeks intervals with 1×10^7^ of the stably CRTAM-FLAG transfected 293 cells. The mab were produced according to standard methods with the SP2/O myeloma cell line. Hybridoma supernatants were tested by flow cytometry on either CRTAM-FLAG transfected or untransfected BWZ.36 cells as described before [21]. For further characterization of chicken CRTAM, we selected mab 8A10 (IgM).

### Cell preparation

Single cell suspensions of bursa, ceacal tonsils, spleen and thymus were prepared by passing the organs through a stainless steel mesh. Lymphocytes were obtained from the cell suspensions by density centrifugation on Ficoll-Hypaque (Biochrom, Berlin, Germany). Peripheral blood lymphocytes (PBL) were isolated by slow-speed centrifugation [22]. Intestinal intraepithelial lymphocytes (IEL) from the duodenal loop were prepared as described before [23].

### Activation of cells and cell culture

Activated lymphocytes isolated from blood, spleen, thymus and caecal tonsils as well as IEL were generated by cultivation 1×10^7^ cells/ml in a 24-well plate with either a 1∶1000 dilution of recombinant IL-2 produced in our laboratory [24] or with plate-bound anti-TCR-2 (SouthernBiotech, Birmingham, USA) at a concentration of 10 µg/ml for different time periods. The cells were cultured with RPMI 1640 medium supplemented with 8% FCS, 2% chicken serum and 1% penicillin/streptomycin at 41°C and analyzed by flow cytometry.

### Antibodies and immunofluorescence analysis

Single-cell staining was performed with the 8A10 mab, followed by a goat-anti-mouse IgM-allophycocyanin (APC) conjugate (SouthernBiotech, Birmingham, USA), or with anti-FLAG M2 mab (Sigma-Aldrich, Munich, Germany), followed by a goat-anti-mouse IgG1 APC (SouthernBiotech, Birmingham, USA) or R-phycoerythrin (R-PE) conjugate (SouthernBiotech, Birmingham, USA), respectively. The staining with the CRTAM-huIg fusion protein was followed by a goat-anti-human IgG R-PE conjugate (SouthernBiotech, Birmingham, USA). For double immunofluorescence analysis, the cells were first incubated with a mixture of primary mab, followed by incubation with an APC-conjugated goat-anti-mouse IgM antibody in combination with goat-anti-mouse IgG1 R-PE conjugate (SouthernBiotech, Birmingham, USA). Additional mab used for staining were specific for chicken CD3 (CT3, mouse IgG1) [25], chicken CD4 and CD8 (CT4, mouse IgG1; CT8 mouse, IgG1 [26], Bu-1 (AV20, mouse IgG1) specific for B lymphocytes [27], CD25 (IgG1), the α-chain of the IL-2-receptor, chicken TCR-1 (mouse IgG1) to discriminate the γδ T cells (γδ-TCR [28]), and TCR-2 and TCR-3 to distinguish different subsets of αβ T cells (αVβ1-TCR [29]; αVβ2-TCR [30], both mouse IgG1). For each staining, appropriate isotype matched controls were used.

Dead cells were discriminated by staining with Fixable Viability Dye eFluor® 780 (affymetrix eBioscience, Frankfurt, Germany) or 7-amino-actinomycin D (7-AAD, Sigma-Aldrich, Munich, Germany; 25 µg/µl) and the living cell population was analyzed by flow cytometry (FACS Canto II, Beckton Dickinson, USA) using the BD FACS DIVA 6.1.3 software.

### BWZ.36 reporter assay and ELISA

The BWZ.36 cells are stably transfected with a ß-galactosidase reporter cassette under the control of the IL-2 promotor containing three NFAT repeats. A construct was generated encoding the extracellular CRTAM domains fused to the CD8 transmembrane domain and the CD3ζ cytoplasmatic domain, which was used to establish a stable BWZ.36 clone. Upon CRTAM ligation the ITAM of the CD3ζ cytoplasmatic domain is phosphorylated and activates the NFAT promotor. As a consequence ß-galactosidase is induced and can be detected by adding chlorophenolred-ß-D-galactopyranosid (CPRG). ß-galactosidase has the ability to cleave CPRG into galactose and chlorophenolred, leading to a color change that can be detected and quantified by optical density reading at 575 nm. 24-well cell culture dishes were coated with anti-FLAG mab 10µg/ml at 4°C over night for a positive control or were left uncoated. Each cell line was plated at a total amount of 1.5×10^5^ cells in the dishes and was incubated for 24 h at 37°C. For our binding study, we co-cultivated stably CRTAM-FLAG transfected BWZ.36 reporter cells with the stably Necl-2-FLAG transfected HEK 293 cells. As positive controls, we incubated the CRTAM-FLAG transfected BWZ.36 in the anti-FLAG mab coated wells. As a mock-control, we co-cultivated the stably expressing BWZ.36 reporter cells with a HEK 293 cell line expressing a CRTAM unrelated molecule to exclude unspecific binding. To verify the specificity of chicken CRTAM to chicken Necl-2 we co-cultivated the Necl-2-FLAG transfected HEK 293 cells with the CRTAM-huIg fusion protein, both pure and in a concentration of 1∶10 for 20 minutes to block specific binding sites before adding the CRTAM-FLAG transfected BWZ.36 cells. After incubation time the cells were lysed and the β-galactosidase activity was measured by using 130 µl/well CPRG (Roche, Mannheim, Germany) and quantified by optical density reading at 575 nm 18 h after incubation.

### Statistical analysis

The data of the BWZ.36 reporter assays is presented as mean value (mean) and standard deviation (SD) and a dependent t-test was employed to assess differences using the IBM SPSS program version 21.0 based on a significance level of 5%. Furthermore an adjustment for multiple testing was done using the Bonferroni-Holm correction for multiple comparisons. Finally, values of p<0.01667 were considered significant.

### Conjugate Assay

Untransfected BWZ.36, mock transfected BWZ.36 and BWZ.36-CRTAM cells were labeled with PKH67 Green Fluorescent Cell Linker Kit for 2 minutes (Sigma-Aldrich, Munich, Germany), whereas 293-Necl-2 cells were labeled with Vybrant® DiD cell-labeling solution (Invitrogen, Karlsruhe, Germany) for 5 minutes using standard procedures. After the cells were washed three times, labeled 293-Necl-2 cells (2,5×10^5^) were mixed with either labeled BWZ.36-CRTAM cells, mock-transfected BWZ.36 cells or untransfected BWZ.36 cells (2,5×10^5^), spun down and incubated at 37°C for 2 h. The conjugates were washed once and gently resuspended before flow cytometric analysis. For blocking experiments CRTAM transfected BWZ.36 cells or mock-transfected BWZ.36 cells were preincubated with 8A10 mab for 20 min at room temperature.

## Results

### Identification of chicken CRTAM

**Figure 1:**
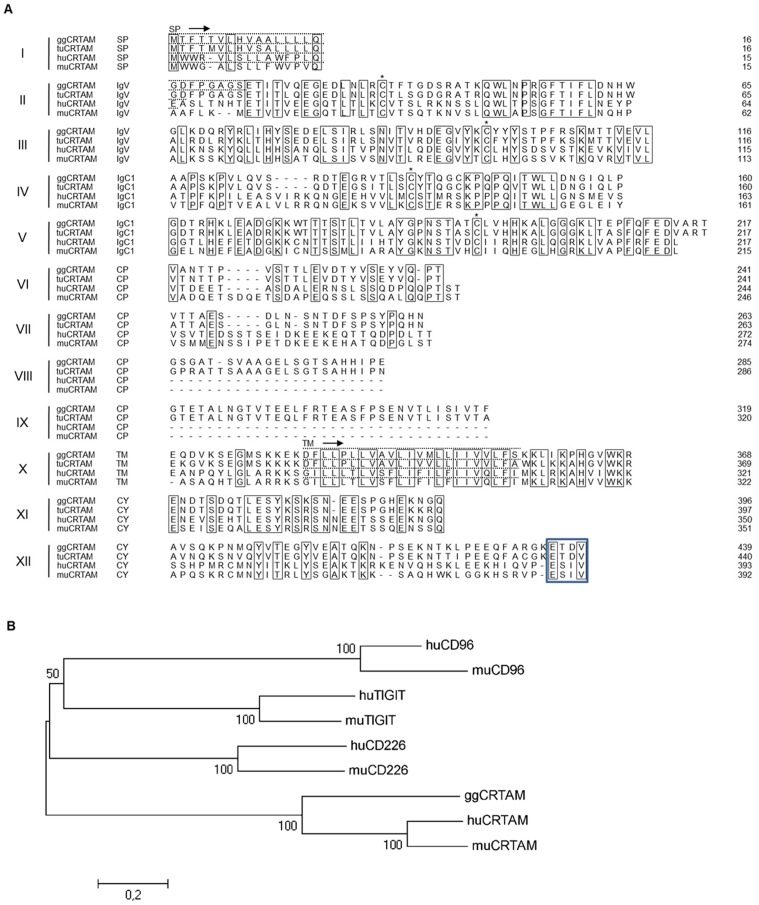
Identification of chicken CRTAM. (A) Ig domains (IgV, IgC1), connecting peptides (CP) and cytoplasmic domains (CY) as well as PDZ-binding motifs (framed) are indicated. The positions of the signal peptides (SP) and the transmembrane domains (TM) are marked by a bar above the sequence. The conserved cysteine residues are marked by asterisks. Exons are numbered according to the genomic structure of the chicken sequence. The alignment was performed separately for the different exons with ClustalW. Identical residues are boxed. (B) In order to delineate the phylogenetic relationships of the chicken and mammalian receptors indicated a neighbor-joining phylogenetic tree was generated using the MEGA5 software. Accession numbers: chicken (gg) CRTAM: NP_001025518.1, turkey (tu) CRTAM: ENSMGAG00000001951, human (hu) CRTAM: NP_062550.2, mouse (mu) CRTAM: NP_062338.3, human CD226: NP_006557.2, mouse CD226: NP_848802.2, human CD96: NP_937839.1, mouse CD96: NP_115854.2, human TIGIT: NP_115854.2, mouse TIGIT: NP_001139797.1.

In an attempt to identify novel Ig-like receptors with immunomodulatory potential we found a previously cloned gene designated cTADS. The gene is located on a 12.5 kb region of chromosome 24 and is encoded by 12 exons. CRTAM sequences can also be found in other avian species (turkey: Chromosome 26: 3,303,411-3,315,820; 12 exons and an incomplete version in zebra finch: chromosome 24:3450462-3458344:1). As depicted in Fig.1A, cTADS shares significant homology with mammalian CRTAM. It contains two Ig domains in its extracellular region, the first represents a V-type and the second a C-type Ig domain. The linking region between the Ig and transmembrane region is extended with more than 50 additional amino acids present in the chicken and corresponding turkey sequence (Fig. 1A). Analysis of the genomic structure revealed the presence of two additional exons in the avian CRTAM genes, responsible for the extended linking region. The cytoplasmic domain is of equal length and contains a C-terminal PDZ-binding motif (Fig. 1A).

We next constructed a phylogenetic tree with the receptors known to bind to Nectins or Nectin-like proteins. As expected, the homologues of human and mouse CD96, TIGIT and CD226 form closely related pairs. The CRTAM receptors form a distantly related clade, but clearly the chicken CRTAM clusters together with its respective mammalian homologues (Fig. 1B). In conclusion, we have identified the chicken cTADS sequence as CRTAM homologue.

### Generation of a chicken CRTAM specific mab that does not react with resting cells

**Figure 2:**
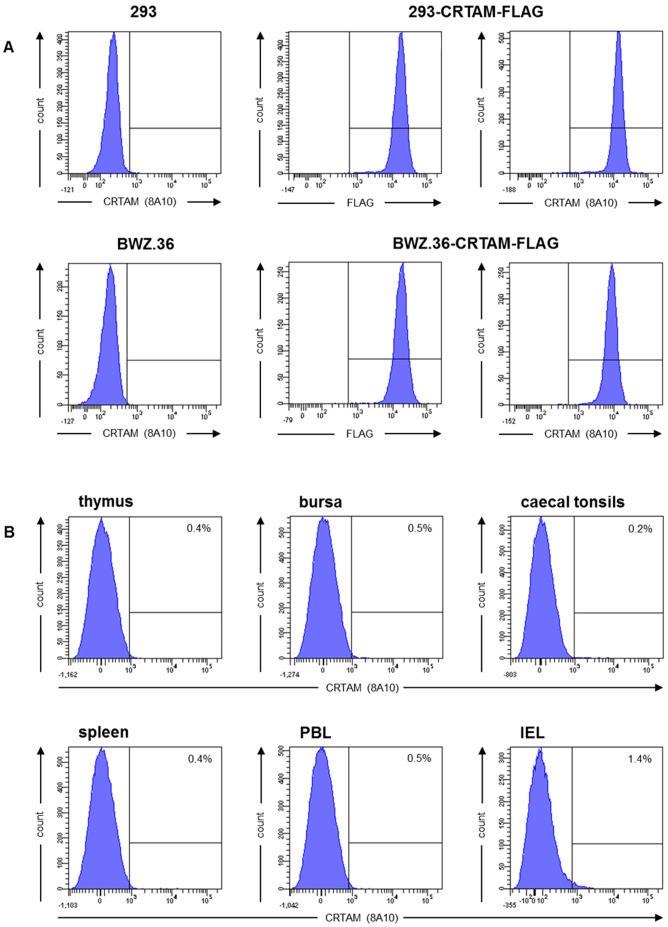
Generation of a chicken CRTAM specific mab. (A) Untransfected and CRTAM-FLAG transfected HEK 293 (upper panel) and BWZ.36 cells (lower panel) were stained with either anti-FLAG or 8A10 mab and analyzed by flow cytometry. (B) Freshly isolated cells of indicated tissues were stained with 8A10 mab. One of three independent experiments is shown.

In order to produce a CRTAM specific mab we generated two cell lines with a N-terminal FLAG-tagged CRTAM version that displayed both extracellular Ig domains fused to the chicken CD8 transmembrane and murine CD3ζ cytoplasmic domains. Following immunization, mab supernatants were evaluated by their binding properties to the stably transfected cell lines in comparison to the positive control FLAG staining and the untransfected controls (Fig. 2A). One mab designated 8A10, which resembles an IgM isotype, was selected for the further studies.

For analyzing the tissue distribution of chicken CRTAM we initially stained cell preparations obtained from thymus, bursa, blood, spleen, caecal tonsils and intestine with the 8A10 mab, however, we failed to detect CRTAM expression on the cell surface of these freshly isolated cells (Fig. 2B). This indicated that the mab is able to specifically recognize CRTAM on transfected cells, but it either did not stain the antigen on native cells or the molecule was not present on these cells.

### CRTAM is transiently expressed on activated cells

Since CRTAM is a receptor known to be upregulated upon activation, we next evaluated if activation of chicken splenocytes would lead to CRTAM expression. Initial experiments were performed with the lectin Concanvalin A, phorbol-myristate-acetate in combination with Ca-ionophore (PMA/Ca-ionophore), recombinant IL-2 and TCR crosslinking (data not shown). While all of the reagents stimulated CRTAM expression, we focused on the latter two reagents due to their superior stimulation of CRTAM.

**Figure 3:**
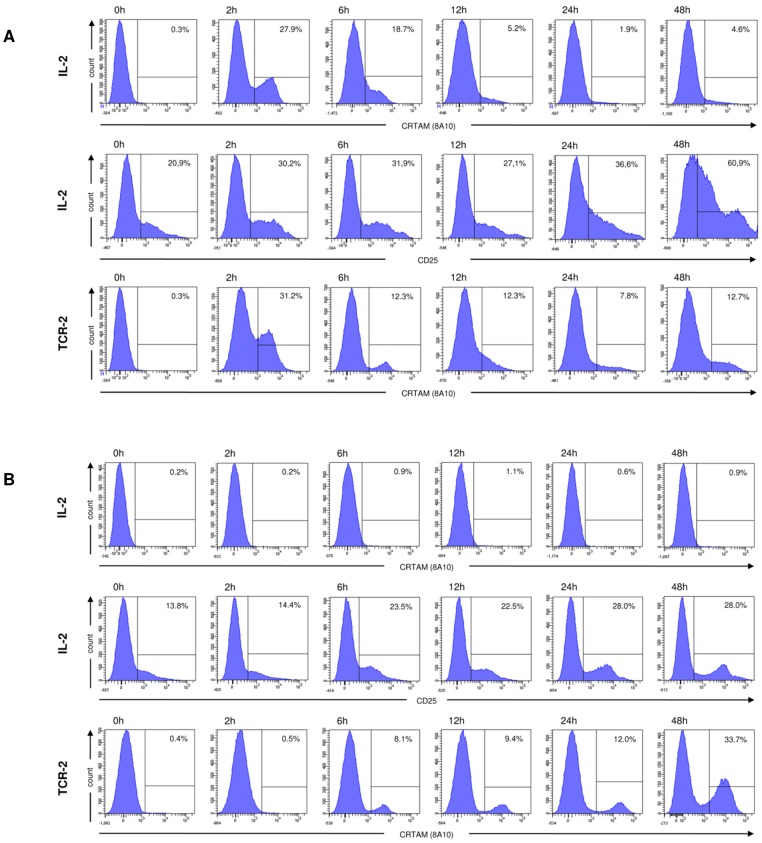
CRTAM expression upon activation. Either splenocytes (A) or PBL (B) were stimulated with IL-2 (upper and middle panels) or plate bound anti-TCR-2 (lower panels) for the indicated time periods followed by immuofluorescence analysis using 8A10 mab and anti-CD25 mab (middle panels). The markers were set according to isotype matched negative controls and the percentage of positive cells is indicated.

IL-2 treatment was able to induce splenocyte activation as judged by increasing CD25 expression (Fig. 3A, middle panels). IL-2 also induced a rapid CRTAM upregulation reaching maximum levels as early as 2 h following stimulation and declining constantly thereafter (Fig. 3A upper panels). Identical treatment of PBL, however, did not induce detectable CRTAM expression (Fig. 3B upper panels) although IL-2 caused cellular activation as judged by the increased CD25 expression during the 48 h stimulation period (Fig. 3B, middle panels). When several dilutions of IL-2 were tested (in the range of 1∶10 to 1∶1000), none of these concentrations were able to induce CRTAM expression (data not shown). Anti-TCR-2 stimulation of splenocytes also induced CRTAM expression after 2 h rapidly declining at later timepoints (Fig. 3A, lower panels). In contrast to the IL-2 stimulation of PBL, that failed to induce detectable CRTAM expression, anti-TCR-2 treatment of PBL resulted in CRTAM expression visible after 6 h and increasing to reach maximum levels at 48 h (Fig. 3B, lower panels).

These results indicate that CRTAM resembles an early activation marker and poses the question what cellular subsets are upregulating CRTAM following activation.

### CRTAM is restricted to CD8 positive T cells with distinct kinetics on αβ versus γδ T cells

**Figure 4:**
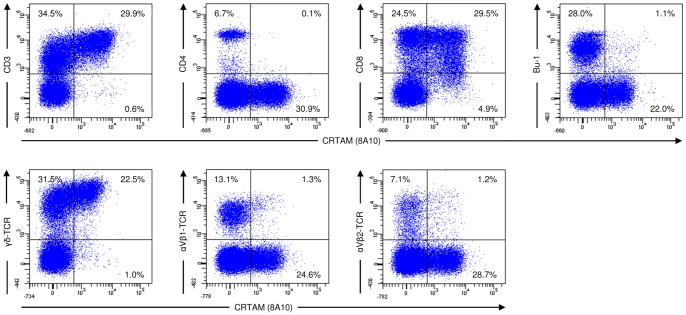
IL-2 stimulation leads to CRTAM upregulation on CD8 positive γδ T cells. Splenocytes were stimulated with recombinant IL-2 for 2 h. Different cell subsets were analyzed by staining with CRTAM specific mab 8A10 in combination with mab specific for CD3, CD4, CD8, Bu1, γδ-TCR, αVβ1-TCR and αVβ2-TCR as indicated. The markers were set according to the isotype matched negative controls. The percentage of positive cells is indicated.

To determine the cell types that express CRTAM we performed a series of double stainings using stimulated splenocytes and PBL. Following short term IL-2 stimulation of splenocytes, CRTAM was detectable on CD3^+^ T lymphocytes. CRTAM was undetectable on CD4^+^ T cells, in contrast it was present on a subset of CD8^+^ T cells (Fig. 4). By analyzing the distinct T cell subsets as characterized by their TCR usage, it became evident, that CRTAM was upregulated on a γδ T cell subpopulation, whereas most αβ T cells did not express CRTAM (Fig. 4). We consistently observed a correlation of TCR and CRTAM expression with those cells expressing high levels of CRTAM also displayed high amounts of TCR on their surface as judged by the fluorescence intensities (Fig. 4).

**Figure 5:**
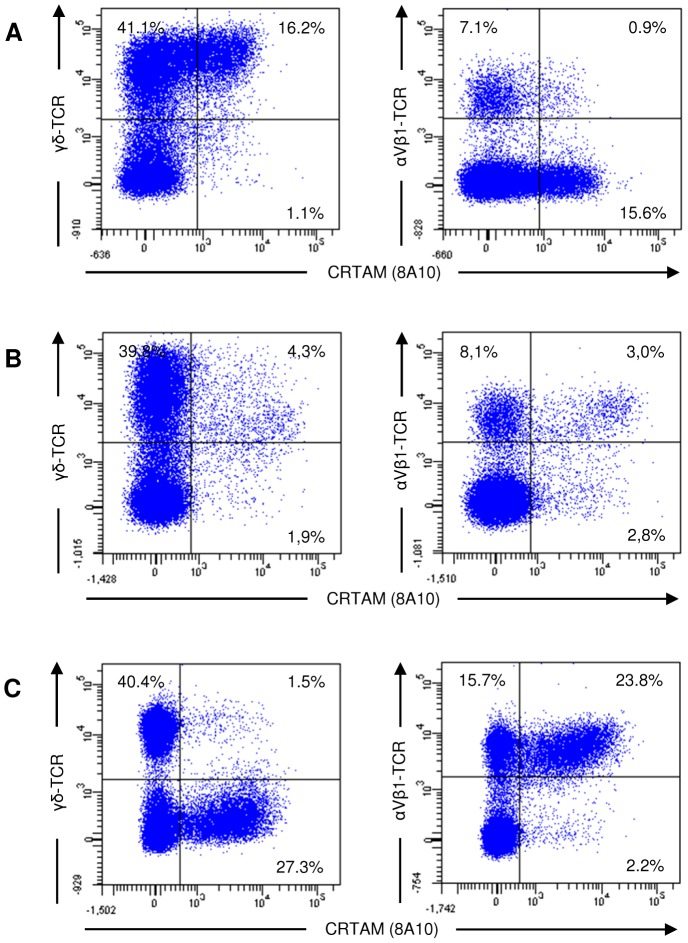
CRTAM expression is induced on splenic γδ T cells and blood αβ T cells at different time points following TCR-2 stimulation. Splenocytes (A, B) and PBL (C) were stimulated with plate bound anti-TCR-2 for 2 h (A) and 48 h (B, C) followed by analysis with mab specific for TCR-γδ or TCR-αVβ1 in combination with 8A10 mab. The markers were set according to the isotype matched negative controls and the percentage of positive cells is indicated. One of three independent experiments is shown.

When similar immunofluorescence analyses were performed with splenocytes activated for 2 h with anti-αVβ1-TCR (TCR-2 mab), CRTAM expression was again mainly expressed by a γδ T cell subset (Fig. 5A). This was surprising since the TCR stimulation was directed against αβ T cells. Only after 24 h to 48 h stimulation where CRTAM expression already decreased, its expression could also be detected on αβ T cells (Fig. 5B). Since we have observed different kinetics of anti-TCR stimulation between splenocytes and PBL, we also analyzed the 48 h time point of stimulated PBL. In that case, CRTAM expression was virtually absent from γδ T cells, but a subset of αβ T cells co-expressed CRTAM (Fig. 5C). These cells were also CD8^+^. The reexpression of CRTAM following TCR-2 stimulation of splenocytes visible in the time period between 24 h and 48 h (Fig. 3A; increase of 8 to 13%) is due to the simultaneous decrease of CRTAM on γδ T cells and its increase on αβ T cells.

In conclusion, depending on the type of stimulation and the cellular source, CRTAM is upregulated on either CD8^+^ γδ or αβ T cells, with the interesting observation that stimulation of the αβ-TCR in splenocytes leads to rapid CRTAM upregulation on γδ T cells

### Identification of the chicken nectin-like 2 molecule

**Figure 6:**
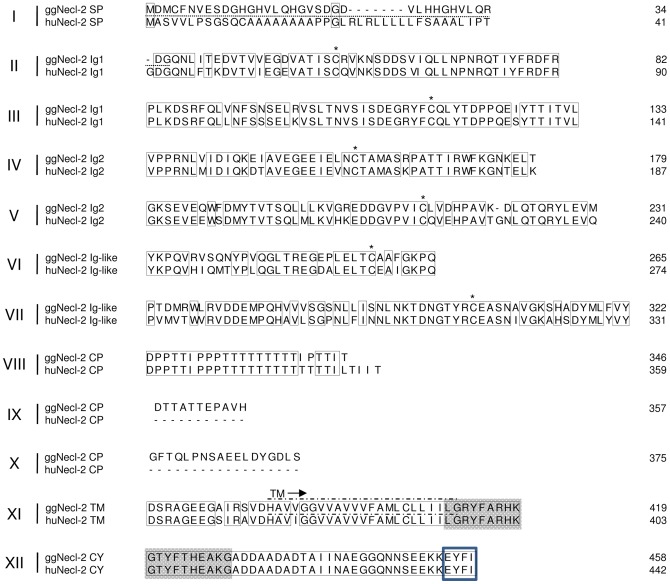
Sequence alignment of chicken (gg) and human (hu) Necl-2. Ig-Domains (Ig1, Ig2, Ig-like), connecting peptide (CP) and cytoplasmic domains (CY) as well as 4.1 (shaded) and PDZ-binding motifs (framed) are indicated. Identical decoration was used as in Fig. 1. Accession numbers: chicken XP_417901, human NP_055148.

To further explore the chicken CRTAM molecule we wanted to test whether it binds to the same ligand as in mammals, which is the nectin-like 2 protein (Necl-2) [5]. For this purpose, the chicken Necl-2 homologue was identified in the genome database by homology searches and cloned by PCR. Chicken Necl-2 is encoded by 12 exons on chromosome 24: 4,664,469-4,732,605 and shares about 80% identity to the corresponding human receptor (Fig. 6). Interestingly, as has been observed for the CRTAM gene, there are two additional exons present in the chicken that code for an extended connecting peptide close to the transmembrane region that is absent from the human sequence (Fig. 6).

As the human counterpart, the intracellular tail of chicken Necl-2 contains a type I PDZ-binding motif (PDZ) characterized by the conserved sequence X/Tyr/Phe/Ile [31] that in humans is able to bind the PDZ motif of members of the MAGUK family, like Pals2 [32], CASK [33] and Dlg3 (MPP3) [34]. Furthermore it also contains a binding motif for band 4.1 family proteins and thereby in humans it is known to recruit the tumor suppressor DAL-1, that connects Necl-2 to the cytoskeleton [35].

### Chicken CRTAM binds to Necl-2

**Figure 7:**
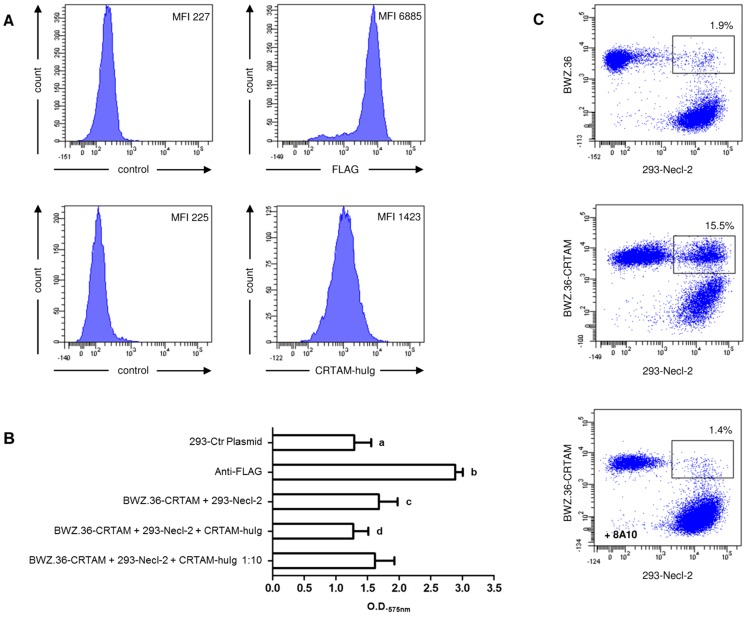
Chicken CRTAM binds to Necl-2. (A) The chicken Necl-2-FLAG transfected HEK 293 cells were stained with either an isotype matched control or anti-FLAG mab (upper panel). The CRTAM-huIg fusion protein was tested on the same cell line (right histogram, lower panel) and compared to a control staining of the Necl-2-FLAG transfected cells with an irrelevant huIg fusion protein (left histogram, lower panel). Mean fluorescence intensity (MFI) is indicated. (B) A reporter assay as described in the methods section was initiated to analyze the binding of CRTAM and Necl-2. Mean ± SD of twelve independent assays is shown. Lower case letters denote significant diferences (p<0.01667) of each denoted group compared with following groups: (a) anti-FLAG positive control (p<0,001), BWZ-CRTAM + 293-Necl-2 receptor ligand pair (p<0,001) and BWZ-CRTAM + 293-Necl-2 receptor ligand pair blocked with 1:10 diluted CRTAM-huIg (p<0,001), (b) all other treatment groups, (c) BWZ-CRTAM + 293-Necl-2 receptor ligand pair blocked with undiluted CRTAM-huIg (p<0,001), (d) BWZ-CRTAM + 293-Necl-2 receptor ligand pair blocked with 1:10 diluted CRTAM-huIg (p<0,001). (C) For conjugate assays, BWZ.36 cells and CRTAM transfected BWZ.36 cells were labeled with PKH67, whereas 293-Necl-2 cells were labeled with Vybrant® DiD. 293-Necl-2 transfectants only formed conjugates with CRTAM transfected (middle panel) but not with untransfected BWZ.36 cells (upper panel). Preincubation of the CRTAM transfected BWZ.36 cells with 8A10 mab prior to coincubation with 293-Necl-2 cells almost completely reduced conjugate formation (lower panel). Percentage of conjugates is indicated. One of three independent experiments is shown.

In order to study the potential interaction of CRTAM and Necl-2 we performed three independent assays. First we generated a soluble CRTAM protein by fusing the two CRTAM Ig domains to the human C_H_2, C_H_3 Ig domains. We tested the specific binding of the CRTAM-huIg fusion protein on a HEK 293 cell line stably transfected with the extracellular N-terminal FLAG-tagged domain of Necl-2 and on untransfected HEK 293 cells, respectively (Fig. 7A). Necl-2 was readily detected by anti-FLAG staining on the transfected HEK 293 cells (Fig. 7A, upper panel; MFI of 6885 as compared to MFI of 227 in the control). Next we stained the cells with the CRTAM-huIg fusion protein. As can be observed from Fig. 7A, the fusion protein stained the Necl-2 transfected cells, whereas an irrelevant fusion protein that also contained the human C_H_2, C_H_3 Ig domains did not react with the Necl-2 transfected cells (Fig. 7A, lower panel; MFI of 1423 as compared to MFI of 225 in the control).In a next step, we verified the binding capacitiy of chicken CRTAM and chicken Necl-2 employing the CRTAM transfected BWZ.36 cell line in a reporter assay. Since the CRTAM was epitope tagged, anti-FLAG stimulation served as a positive control and resulted in a strong induction of the intracellular NFAT promotor and ß-galactosidase activity (Fig. 7B). The BWZ.36-CRTAM transfectants were either incubated with HEK 293 cells expressing an irrelevant receptor as a negative control or with the Necl-2 expressing HEK 293 cells. The Necl-2 expressing cells induced significant higher amounts of ß-galactosidase activity as compared to the control (Fig. 7B). This interaction could be blocked by the addition of the soluble CRTAM-Ig fusion protein.

The statistical analysis revealed significant differences (p<0,01667) between all groups except the negative control compared to BWZ-CRTAM + 293-Necl-2 receptor ligand pair blocked by CRTAM-huIg and BWZ-CRTAM + 293-Necl-2 receptor ligand pair to BWZ-CRTAM + 293-Necl-2 receptor ligand pair blocked by 1∶10 diluted CRTAM-huIg. As measure of the variability we calculated subject coefficients of variation, which all were<10% indicative of a good reproducibility. The calculation of the minimum distance in between the value of the negative control and the value of the BWZ-CRTAM + 293-Necl-2 receptor ligand pair is based on the largest statistical variation. As a result we can assign, that the value of the BWZ-CRTAM + 293-Necl-2 receptor ligand pair has to exceed the value of the negative control more than 20% to ensure that the ligand binds to the receptor with a certainty of 95%. In this assay the value of BWZ-CRTAM + 293-Necl-2 receptor ligand pair exceeds more than 30% compared to the value of the mock-transfected negative control.

In a further experiment we coincubated labeled BWZ.36-CRTAM, or control BWZ.36 cells with 293-Necl-2 cells for 2 h at 37°C and measured the formation of conjugates by flow cytometry (Fig. 7C) We observed abundant conjugate formation between BWZ.36-CRTAM and 293-Necl-2 cells (Fig. 7C, middle panel) but not between untransfected BWZ.36 (Fig. 7C, upper panel) or mock transfected BWZ.36 (data not shown) and 293 Necl-2 cells. Preincubation of BWZ.36-CRTAM cells with 8A10 mab almost completely blocked the conjugate formation (Fig. 7C, lower panel), whereas preincubation of mock transfected BWZ.36 cells with the 8A10 mab did not show any effect (data not shown).

In conclusion, three independent assays have proved the CRTAM - Necl-2 interaction.

## Discussion

The chicken cTADS gene was initially identified in 2000 as a novel Ig-like molecule with similarity to several proteins mediating adhesion that is upregulated on activated thymocytes and splenocytes [10]. The mammalian CRTAM gene was identified in 2000, too, thus a direct assignment of CRTAM and cTADS was most likely precluded due to the simultaneous submission [3]. In 2004, a study initiated to establish a link between innate immunity in the urochordate Ciona intestinalis and adaptive immunity receptors of the IgSF family pointed out the relationship of mammalian CRTAM and cTADS [36]. It is of interest that CRTAM homologues seem to have distantly related homologues not only in non-mammalian vertebrates but even in Drosophila, where the BEAT family shares similar structural features such as an identical domain organization [36]. In Ciona intestinalis, an ancestral gene cluster represented on chromosomes 4 and 10 founded four paralogous regions by two whole genome duplications [37]. Comparative studies have identified the chicken chromosomes 1, 4, 24 and 31 as these paralogous regions and indeed several important IgSF families have been located to these chromosomes, such as the CD200 family on chromosome 1, the chicken leucocyte receptor cluster (LRC) on chromosome 31, and CRTAM on chromosome 24 [12], [16], [38].

It is interesting to note that both chicken Necl-2 and CRTAM have a difference in their genomic organization. There are two additional exons coding for residues located between the membrane proximal Ig domains and the transmembrane domains. It is not evident, why this region has been condensed in the mammalian molecules and what the functional significance of an extended membrane proximal region is. In a recent report, a novel computational method was used to demonstrate the close relationship of CRTAM and the nectin-like family with the conclusion that both receptors belong to the nectin-like family [39]. The finding that both genes share the additional exons in galliformes is supporting this notion. A further unifying theme for CRTAM and other nectins is their expression on leukocytes and on cells of the nervous system such as Purkinje cells in the cerebellum [40]. The analysis of CRTAM expression in the brain goes beyond the scope of this report, but will be examined in future studies. Altogether, CRTAM is a well conserved molecule that serves several functions in the immune and nervous system.

In order to further explore chicken CRTAM on leukocytes we have generated a specific mab. Our inability to detect CRTAM on freshly isolated cells corresponds well with mammalian data on CRTAM expression. Several activation agents such as mitogens, TCR crosslinking and IL-2 rapidly upregulated chicken CRTAM. One intriguing aspect is the fast CRTAM upregulation within 2 h of IL-2 or TCR stimulation of splenocytes. This is in good agreement with the data reported on the mRNA expression of cTADS [10]. Since mRNA is detectable very early after cellular activation, it is likely to resemble de novo protein biosynthesis of CRTAM rather than surface expression of preformed receptor proteins stored intracellularly as reported for other molecules such as CD300a [41].

Interestingly, this early CRTAM upregulation on splenocytes by IL-2 was mainly detectable on a γδ T cell subset. IL-2 stimulation of PBL failed to induce CRTAM, although the cytokine was able to activate cells as judged by the increase of CD25 expression. In contrast, TCR crosslinking did induce CRTAM expression on PBL, but at later time points with a maximum after 48 h of stimulation and mainly on αβ T cells.

Taken together, there are two pronounced differences in CRTAM upregulation between γδ and αβ T cells: firstly, a rapid upregulation on γδ T cells upon stimulation and a delayed CRTAM expression on αβ T cells reaching a maximum within 48 h and secondly an inability of blood derived γδ T cells to express CRTAM in contrast to splenic γδ T cells. Splenic and blood derived γδ T cells also differ in their expression of various other molecules, such that blood γδ T cells lack CD6 and display only low levels of CD5 [42]–[44]. Thus, while blood γδ T cells seem to be refractory to activation, a splenic γδ T cell subset can be readily activated and CRTAM serves as an early activation antigen on these cells. In this regard it is interesting that another rich source of γδ T cells is the IEL population, however, we were not able to detect CRTAM on resting or activated IEL. This underscores that IEL harbors yet another γδ T cell subset, that is also defined by its unique expression of CD8αα homodimers instead of CD8αβ heterodimers [45].

Mammalian CRTAM is also detectable on NK cells. In the adult chicken, NK cell frequencies are either low or undetectable in several tissues such as PBL or spleen, but the intestinal epithelium harbors a high frequency of NK cells [46], [47]. This IEL population did not express CRTAM whether on the resting cell population, nor were we able to increase CRTAM expression upon stimulation of IEL with either IL-2, TCR-2 crosslinking, or PMA/Ca-ionophore (data not shown).

A further interesting result of our study is the early upregulation of CRTAM on γδ T cells following stimulation of αβ T cells with TCR crosslinking. We can exclude a direct stimulation, since multiple studies have demonstrated that the TCR-2 mab is specifically recognizing αVβ1 T cells. For instance, staining with the TCR-1 and TCR-2 mab detects non-overlapping T cell subsets [48] and immunoprecipitation with these mab also shows distinct biochemical features [49]. Therefore it could be speculated, that stimulation of αβ T cells leads to either a cognate or non-cognate interaction of αβ and γδ T cells that induces CRTAM on γδ T cells. IL-2 secreted by αβ T cells may well be involved in this process, since IL-2 by itself causes CRTAM expression on γδ T cells. This observation is in line with earlier reports showing that γδ T cells in the chicken require either αβ T cells or exogenous cytokines as growth promoting factors [50]. Since assays for the detection of chicken IL-2 are not available, we performed an IL-2 specific PCR on either resting or TCR-2 stimulated splenocytes. In this experiment a strong upregulation of IL-2 mRNA is observed (Fig. S1, Text S1). The mechanism of CRTAM induction on γδ T cells following TCR-2 crosslinking needs to be studied in greater detail in future experiments. This will also include an analysis of CD25 expression on resting chicken T cells, which has so far not been performed comprehensively.

We have also been able to confirm Necl-2 as ligand for CRTAM. In mammals, Necl-2 is expressed on the basolateral membrane of epithelial cells and on certain tumor cells [2], [7]. The interaction of CRTAM and Necl-2 leads to IFN-γ secretion and T cell stimulation as well as secretion of IL-22 [2]. It will be of importance to clarify if similar reactions can be induced in the chicken. Likewise, the recent observation that CRTAM engagement on Vγ9Vδ2 T cells triggers cell death of γδ T cells could indicate that the upregulation of CRTAM on splenic γδ T cells in the chicken is a sign of terminal differentiation that may finally result in apoptosis if CRTAM binds to Necl-2 [8].

In conclusion, this characterization of chicken CRTAM expression revealed that CRTAM belongs to a highly conserved receptor family interacting with nectins and that it resembles an activation antigen differentially regulated on splenic versus blood αβ and γδ T cells.

## Supporting Information

Text S1 Supplementary Materials and Methods.
(TIF)
