# Correction: Chicken CRTAM Binds Nectin-Like 2 Ligand and Is Upregulated on CD8^+^ αβ and γδ T Lymphocytes with Different Kinetics

**DOI:** 10.1371/annotation/22ed1b95-740d-4308-89c5-770a24375b74

**Published:** 2014-01-02

**Authors:** Maria Zechmann, Sven Reese, Thomas W. Göbel

A figure file was erroneously omitted from the list of Supporting Information. The publisher apologizes for the error. The figure's description is "Detection of a splenic IL-2 transcript after 2 h of TCR-2 stimulation," and it can be viewed here: 
