# Supplementary figures and images for: Chicken CRTAM Binds Nectin-Like 2 Ligand and Is Upregulated on CD8+ αβ and γδ T Lymphocytes with Different Kinetics
Source: PLoS One. 2013 Dec 10;8(12):e81942. doi: 10.1371/journal.pone.0081942 (PMC3858274; doi:10.1371/journal.pone.0081942)

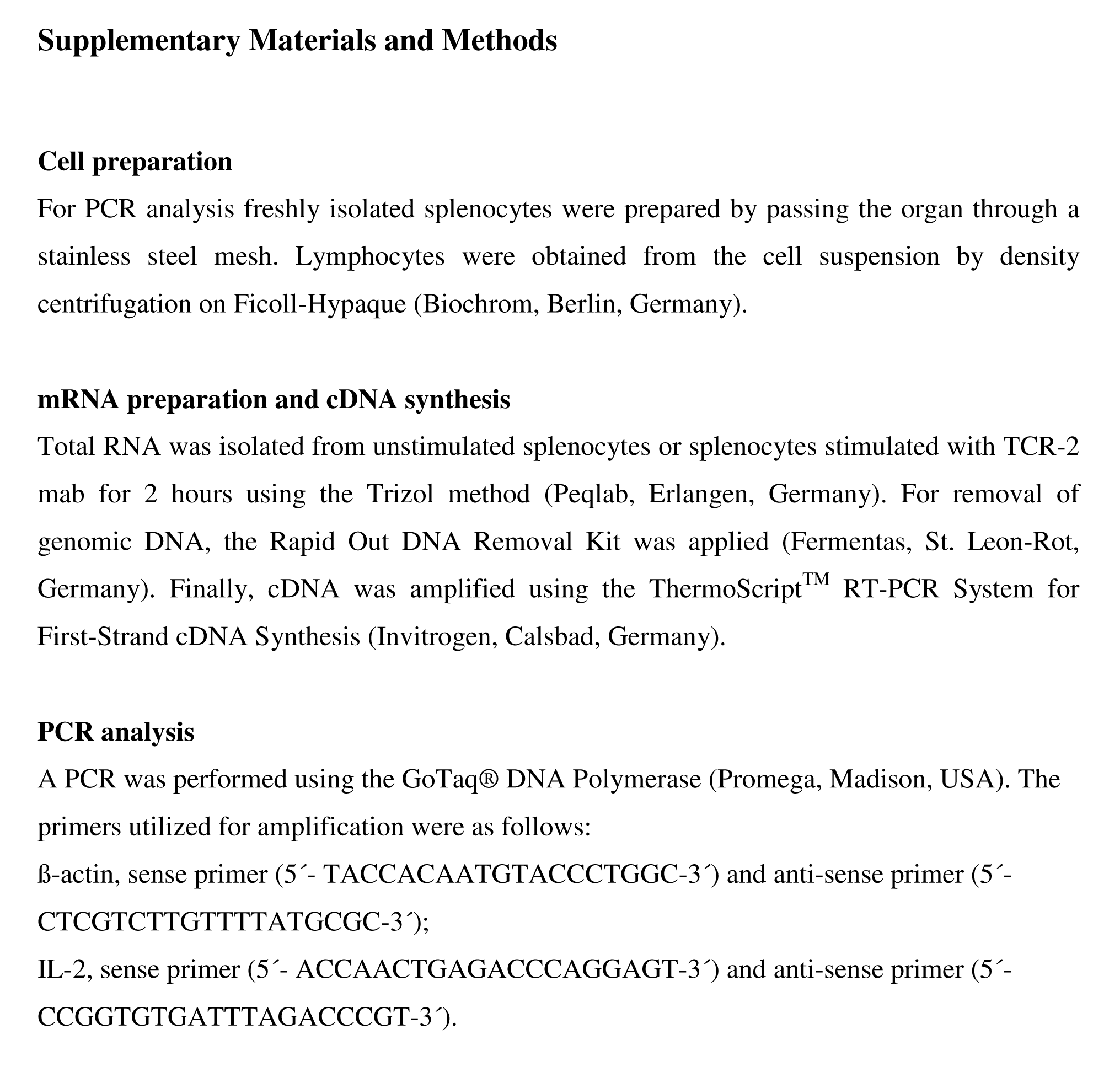

Supplement: Text S1 — Supplementary Materials and Methods. (TIF) [file pone.0081942.s001.tif]
