# Supplementary figures and images for: Correction: Chicken CRTAM Binds Nectin-Like 2 Ligand and Is Upregulated on CD8+ αβ and γδ T Lymphocytes with Different Kinetics
Source: PLoS One. 2014 Jan 2;9(1):10.1371/annotation/22ed1b95-740d-4308-89c5-770a24375b74. doi: 10.1371/annotation/22ed1b95-740d-4308-89c5-770a24375b74 (PMC3880370; doi:10.1371/annotation/22ed1b95-740d-4308-89c5-770a24375b74)

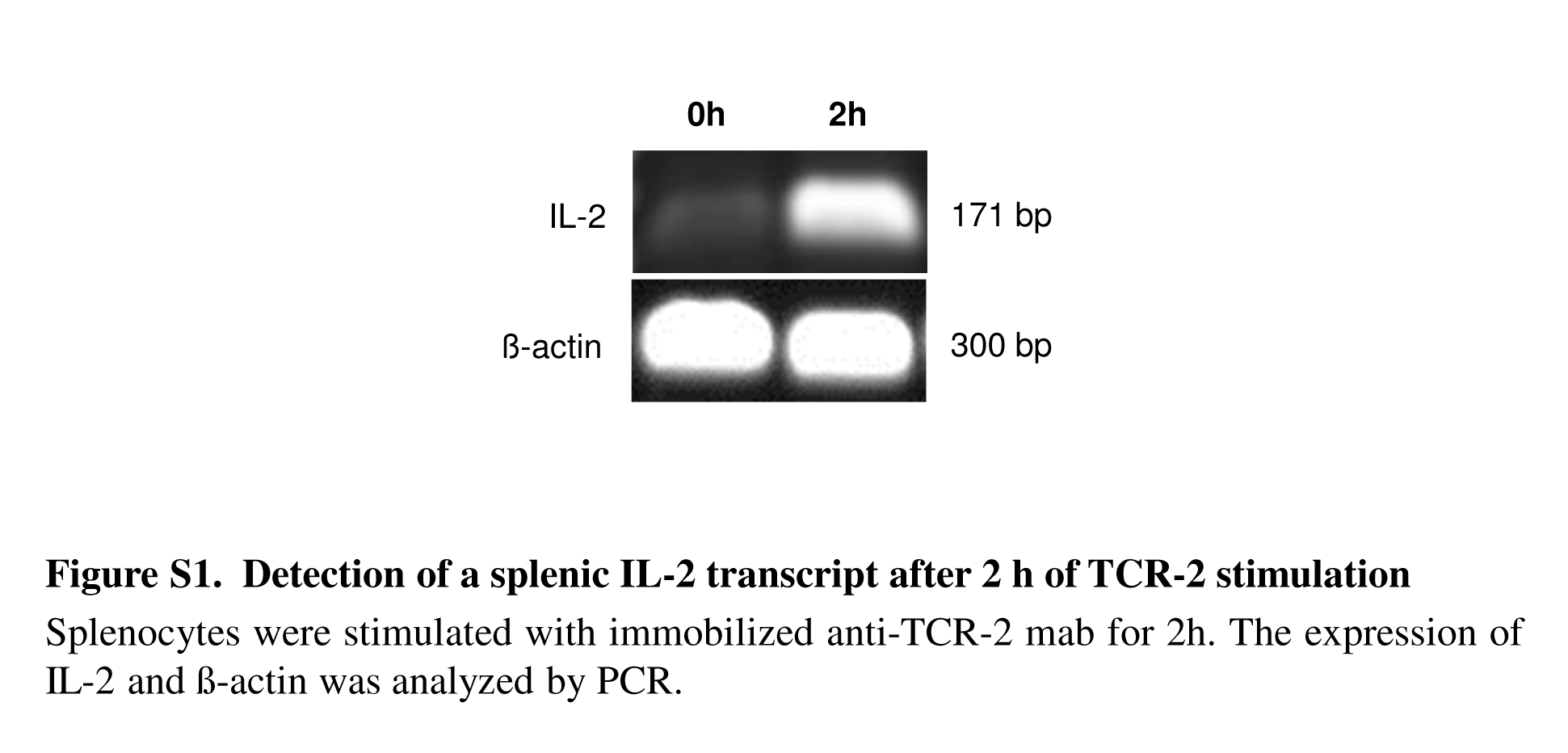

Supplement: Supplementary file 1 [file pone.22ed1b95-740d-4308-89c5-770a24375b74.s001.tif]
